# The low molecular weight fraction of compounds released from immature wheat pistils supports barley pollen embryogenesis

**DOI:** 10.3389/fpls.2015.00498

**Published:** 2015-07-07

**Authors:** Rico Lippmann, Swetlana Friedel, Hans-Peter Mock, Jochen Kumlehn

**Affiliations:** Leibniz Institute of Plant Genetics and Crop Plant Research (IPK)Gatersleben, Germany

**Keywords:** co-culture, feeder signals, haploid technology, microspore, nurse culture, plant-regeneration

## Abstract

Pollen embryogenesis provides a useful means of generating haploid plants for plant breeding and basic research. Although it is well-established that the efficacy of the process can be enhanced by the provision of immature pistils as a nurse tissue, the origin and compound class of the signal molecule(s) involved is still elusive. Here, a micro-culture system was established to enable the culturing of populations of barley pollen at a density too low to allow unaided embryogenesis to occur, and this was then exploited to assess the effect of using various parts of the pistil as nurse tissue. A five-fold increase in the number of embryogenic calli formed was obtained by simply cutting the pistils in half. The effectiveness of the pistil-conditioned medium was transitory, since it needed replacement at least every 4 days to measurably ensure embryogenic development. The differential effect of various size classes of compounds present in the pistil-conditioned medium showed that the relevant molecule(s) was of molecular weight below 3 kDa. This work narrows down possible feeder molecules to lower molecular weight compounds and showed that the cellular origin of the active compound(s) is not specific to any tested part of the pistil. Furthermore, the increased recovery of calli during treatment with cut pistils may provide a useful tool for plant breeders and researchers using haploid technology in barley and other plant species.

## Introduction

Cell-to-cell communication is a fundamental requirement for cell proliferation in all multicellular organisms. Some signaling mechanisms involve adjacent cells, but others act over substantial distances. Plant compounds reminiscent of mitogens (a well-researched set of animal long-distance signaling compounds), or at least displaying signal transducer activity, have been identified. While some of these signaling molecules have proven to be small metabolites, others are polypeptides and other complex molecules. The role of phytohormones (e.g., auxins and cytokinins) in the regulation of cell division and differentiation has been exhaustively researched (Lindsey et al., 2002), while certain sugars have also been shown to participate in the control of cell division (Riou-Khamlichi et al., 1999; Eveland and Jackson, 2012). Various proteins like chitinases, peroxidases, or arabinogalactan proteins have all been shown to have a signaling function during embryogenesis, pollen tube differentiation and root development (De Jong et al., 1992; Van Engelen and de Vries, 1992; Wink, 1994; Willats and Knox, 1996). In the last decade however, peptides have been increasingly recognized to act as growth factors and as cell-to-cell signaling molecules (Lindsey et al., 2002). An example is the pentapeptide phytosulfokine alpha, which has been shown to promote cell division in asparagus cell suspension and rice protoplast cultures, somatic embryogenesis in carrot (Matsubayashi et al., 2001) as well as pollen embryogenic development in *Triticeae* species (Asif et al., 2014). Triggering plant cell division clearly requires the perception of certain extracellular signals (Stuart and Street, 1971; McCabe et al., 1997), as does embryo formation in *Fucus* sp. (Berger et al., 1994). Auxin, in conjunction with particular cell wall components, provides the signaling required to co-ordinate polarity establishment (Souter and Lindsey, 2000). The full nature of the plant signaling network however remains far from being fully understood.

The general importance of cell-to-cell communication is well-illustrated by the observation that in a suspension cell culture, a threshold population density (commonly at least 10,000 per mL) exists, below which cell division is not sustained (Spangenberg and Koop, 1992). Vrinten et al. (1999) demonstrated that embryogenic development is significantly reduced when a density of 10^4^ isolated barley microspores per mL is used instead of 10^5^. In some situations, achieving a certain threshold is sufficient to maintain cell division and development (Shillito et al., 1983; Hoekstra et al., 1993), but in others, there is a further requirement to supply heterologous materials as a nurse tissue (Zheng et al., 2002). The use of nurse tissue has proven to be particularly beneficial in attempts to culture isolated somatic or gametophytic cells and protoplasts. Some research effort has been devoted to establishing culture conditions in which cell density dependence can be removed; typically, this has involved the formulation of complex media, the co-cultivation of nurse material, or the use of micro-cultures (reviewed by Spangenberg and Koop, 1992).

Haploid technology provides a rapid means of fixing meiotic recombination, and is used in a growing number of crop improvement programs. One of the major bottlenecks which has been encountered is species and genotypic variation with respect to the efficiency of inducing *in vitro* cultured pollen to become embryogenic. Embryogenic development of immature barley pollen can be triggered by a temporary impact of stress conditions such as low or high temperatures or starvation (Daghma et al., 2012). The process is then initiated by a symmetric cell division of either the microspore or the vegetative cell of bicellular pollen. This deviation from regular pollen development is followed by successive rounds of cell divisions initially taking place within the pollen envelope (González-Melendi et al., 2005; Daghma et al., 2014). Right after pollen wall rupture that is caused by the exponentially proliferating cells, the calli do typically not show any tissue differentiation. At this developmental stage, some particularly spherical individuals could at best be interpreted as embryo proper-like structures. Yet soon thereafter, the majority of calli form a smooth, periderm-like surface and some assume a pear-shaped form reminiscent of immature zygotic embryos, which reveals the highly embryogenic nature of pollen-derived calli (Oleszczuk et al., 2006). The typical eventual outcome of further development is that multiple embryo-like structures are formed at the callus surface, much like somatic embryos that can be produced from the scutellum of cultured immature zygotic embryos.

The provision of dissected immature barley pistils was shown by Köhler and Wenzel (1985) to enhance the success rate of pollen embryogenesis, and this finding has since been widely exploited in the production of doubled haploids in barley (Li and Devaux, 2001; Lu et al., 2008), wheat (Zheng et al., 2002), oilseed rape (Huang et al., 1990), and maize (Szarka et al., 2001). A comparison of the benefit of including various immature flower explants in wheat has shown that it is the pistil which is the most effective in stimulating embryogenic development (Puolimatka and Pauk, 1999). As wheat pollen embryogenesis appears to be scarcely possible without recourse to nurse tissue, the inclusion of dissected pistils has long become the standard practice for embryogenic pollen cultures (EPCs) of this species.

In a previous attempt to identify substances responsible for the stimulatory effect of conditioned media, Köhler and Wenzel (1985) used a culture method that allows pollen to be released from cultivated immature anthers into liquid culture medium pre-conditioned by dissected barley pistils. Whereas, proteins, amino acids, and sugars were experimentally excluded to possess a conditioning effect, a lower-molecular weight organic substance was demonstrated to stimulate microspore-derived callus production. Its retardation in thin-layer chromatography was very similar to that of indoleacetic acid, however, the substance was shown not to contain an indole component and the true nature of this substance eventually remained elusive to date.

Vrinten et al. (1999) identified genes specifically expressed at the onset of embryogenic development of isolated barley microspores. Two of these genes (*HvECGST* and *HvECLTP*) encode secreted proteins that may be involved in the protection of cells from damage by reactive oxygen species which are assumed to excessively occur as a consequence of stress treatment required to induce embryogenic pollen development. However, the most interesting candidate gene found in the same study was named *ECA1* (early culture abundant 1) and codes for another secreted protein with similarity to the protein component of a hydroxyproline-deficient arabinogalactan protein (AGP) previously detected in suspension-cultured cells of carrot (Baldwin et al., 1993). In addition, *HvECA1* expression was shown to be significantly reduced in low-density cultures in which embryogenic development is hampered. By analysing molecules secreted from EPCs of barley and maize, respectively, Paire et al. (2003) and Borderies et al. (2004) confirmed the presence of AGPs, which further supported the concept that these glycoproteins may belong to the substances essentially involved in the requirement of a cell culture density threshold as well as in the beneficial effect of conditioned media. Later, Letarte et al. (2006) demonstrated that the addition of the arabinogalactan Larcoll or the AGP gum arabicum to EPCs of wheat reduced microspore mortality and increased the number of embryogenic structures produced.

Here, we have developed a barley pollen micro-culture system in an attempt to identify the origin of the stimulatory molecule(s) secreted by the immature wheat pistil and parts thereof and reduce possible compound classes. The density of pollen was reduced below the normal threshold for embryogenesis to be initiated in the absence of co-cultivated wheat pistils, and the nurse tissue itself was dissected to determine which part of the pistil produced the stimulatory effect. A pistil-conditioned medium was then used to specify both the size and stability of the stimulating molecule(s).

## Results

### The effect of various pistil parts on pollen embryogenic development

**Figure 1:**
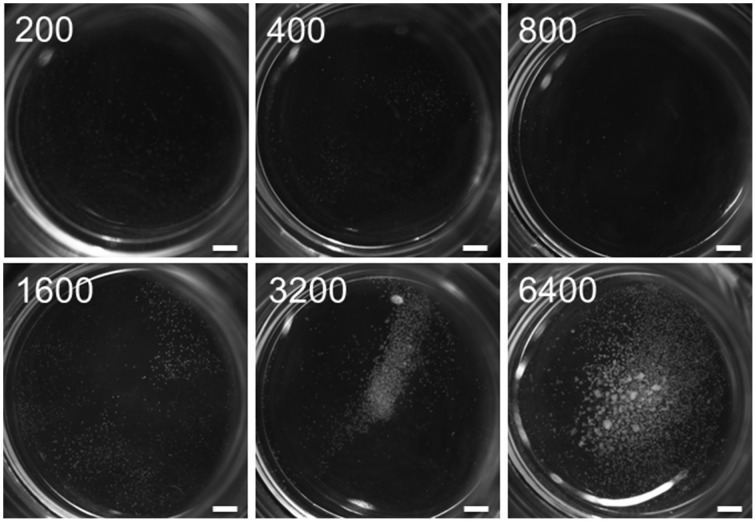
**Threshold of self-feeder effect in embryogenic pollen culture**. Isolated microspores of cv. “Igri” were cultured at various densities (numbers per mL are given on the individual photographs recorded after 4 weeks of culture) in the absence of nurse tissue to determine the minimum culture density required to permit embryogenic development. The microspores were kept in Millicell inserts to facilitate the observation of their response at low culture density, while the inserts were positioned in 3.5 cm Petri dishes containing a total volume of 2 mL medium. Callus formation was completely inhibited at a culture density below the threshold level of ca. 1000 microspores per mL medium, whereas embryogenic calli developed only in cultures with a density of 3200 mL or higher. Bar size = 1.5 mm.

**Figure 2:**
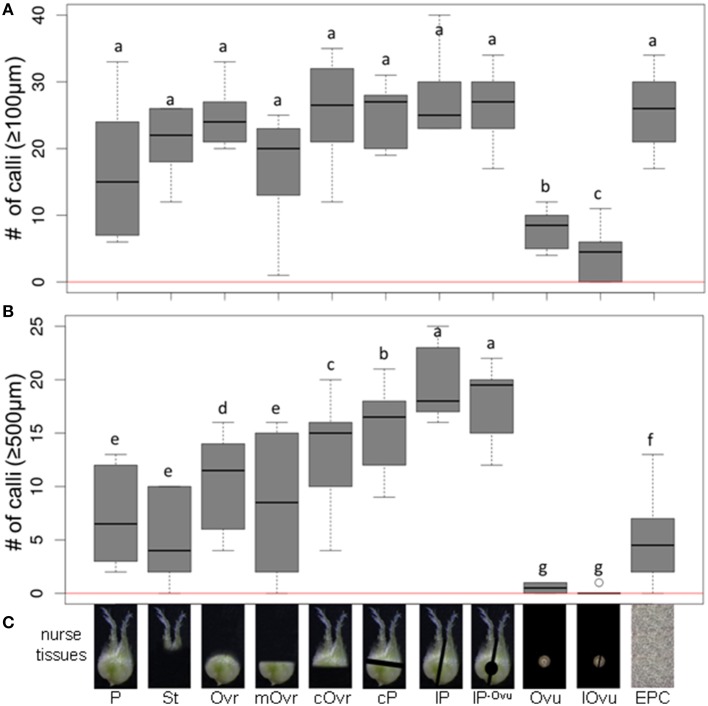
**The effect of whole or partial immature wheat pistils used as a nurse tissue on embryogenic pollen development**. Isolated microspores at an initial density of 75 per mL were co-cultivated with various nurse tissues. The microspores were kept in Millicell inserts to facilitate the observation of their response at low culture density and without being obscured by nurse tissue. The inserts were positioned in 3.5 cm Petri dishes containing a total volume of 2 mL medium, while the nurse tissue was kept in the medium portion outside the inserts. The diagrams show the formation of embryogenic calli of diameter **(A)** ≥100 μm and **(B)** ≥500 μm after 4 weeks of culture. **(C)** Nurse tissues used. Three entire pistils or parts thereof were used per mL medium. P, pistil; St, stigma; Ovr, ovary; mOvr, mycropylar ovary half; cOvr, chalazal ovary half; cP, cross-bisected pistil; lP, longitudinally bisected pistil; lP^-Ovu^, longitudinally bisected pistil without ovule; Ovu, ovule; lOvu, longitudinally bisected ovule; EPC, embryogenic pollen culture of cv. “Igri” precultured for 1–2 weeks and used as nurse tissue at a culture density of 50,000 pollen grains per mL medium. The red horizontal lines represent the responses of respective cultures in the absence of nurse tissue.

The minimum culture density of immature pollen grains of cv. “Igri” required to permit embryogenic development without the need of any nurse tissue was 3200 per mL (Figure 1); lower densities than this supported only a few rounds of cell division, insufficient to produce any regenerable callus. In assessing the stimulatory effect of the nurse tissue therefore, the culture density was reduced by more than one order of magnitude to 75 grains per mL. The nurse tissues tested consisted of either entire or specific parts of wheat pistils (Figure 2), and all of these induced the formation of small calli (100–500 μm) to a similar extent (Figure 2A), while the negative control (no nurse tissue) did not develop any callus (Supplemental Table S1). Isolated ovules (both longitudinally bisected and uncut; lOvu, Ovu) had much the weakest stimulatory effect on callus growth, which was diagnosable already after 7 days of culture due to the comparatively low proportion of enlarged pollen (Supplemental Figure S1). After 2 weeks of culture, the pollen co-cultivated with stigmas (St), ovules, whole ovaries (Ovr; pistil with the stigma being cut off) or the basal, micropylar section thereof (mOvr) remarkably lagged behind in development as compared to those supported by the other tested nurse materials that had already stimulated the formation of larger individuals reminiscent of immature embryos (Supplemental Figure S1). A callus diameter of at least 500 μm is generally considered as being large enough to be capable of whole plant regeneration. Supporting the test pollen with an EPC at a density of 50,000 per mL induced the formation of only a small number of calli ≥500 μm; nonetheless, this positive control treatment was significantly superior to the co-cultivation of ovules or to pollen cultured without nurse tissue (Figure 2B, red line). While the stigma or the micropylar section of the ovary could satisfactorily substitute for the whole pistil (P) as a nurse tissue, longitudinally bisected pistils (lP) were superior to perpendicularly (cross-)bisected ones (cP), which were in turn superior to the chalazal part of the ovary (cOvr); and the latter was superior to the whole ovary (Figures 2B,C). Taken together, bisected pistils were consistently superior to other tested nurse tissues in supporting the formation of embryogenic calli ≥500 μm, whereas the behavior of longitudinally bisected pistils, either with (lP) and without (lP^−Ovu^) ovular tissue attached, showed that the presence of gametophytic tissue made no positive contribution to the stimulatory effect of the pistil. The relevant raw data are presented as Supplemental Table S1, and a representative image set during the cultivation of embryogenic pollen is given as Supplemental Figure S1.

**Figure 3:**
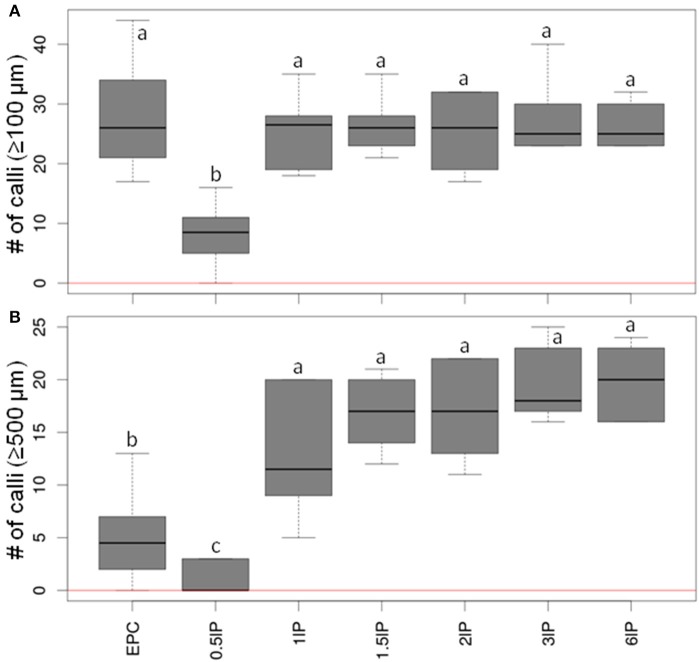
**The effect of varying the number of co-cultivated, longitudinally bisected pistils on embryogenic pollen development**. Isolated microspores at an initial density of 75 per mL were co-cultivated with various numbers of bisected wheat pistils. The microspores were kept within Millicell inserts to facilitate the observation of their response at low culture density and without being obscured by nurse tissue. The inserts were positioned in 3.5 cm Petri dishes containing a total volume of 2 mL medium, while the nurse tissue was kept in the medium portion outside the inserts. The diagrams show the formation of embryogenic calli of diameter **(A)** ≥100 μm and **(B)** ≥500 μm after 4 weeks of culture. EPC, embryogenic pollen culture of cv. “Igri” precultured for 1–2 weeks and used as nurse tissue at a density of 50,000 pollen grains per mL; lP, longitudinally bisected pistil (numbers of bisected pistils co-cultivated per mL medium are given). The red horizontal lines represent the responses of respective cultures in the absence of nurse tissue.

Since the presence as a nurse tissue of longitudinally bisected pistils gave the strongest stimulatory effect on embryogenic pollen development, an attempt was then made to optimize the number of pistils to be used. To this end, low-density cultures (75 isolated microspores of cv. “Igri” per mL medium) involving the co-cultivation of 0.5–6 bisected pistils and EPCs at standard density as positive control were compared. The inclusion of between one and six longitudinally bisected pistils per mL medium was the most effective treatment (Figure 3; raw data given in Supplemental Table S2). The median values of callus formation ≥500 μm in size increased step-wise with the number of co-cultivated pistil halves, while the use of only half a pistil per mL resulted in a significantly reduced pollen-derived callus formation as compared with all treatments involving one to six bisected pistils. The response across the tested range of numbers of pistils added to the cultures indicated that just one bisected pistil is capable of almost entirely satisfying the demand for stimulating signal molecules under the given circumstances.

### Pollen embryogenesis and plant regeneration in cv. “golden promise”

**Figure 4:**
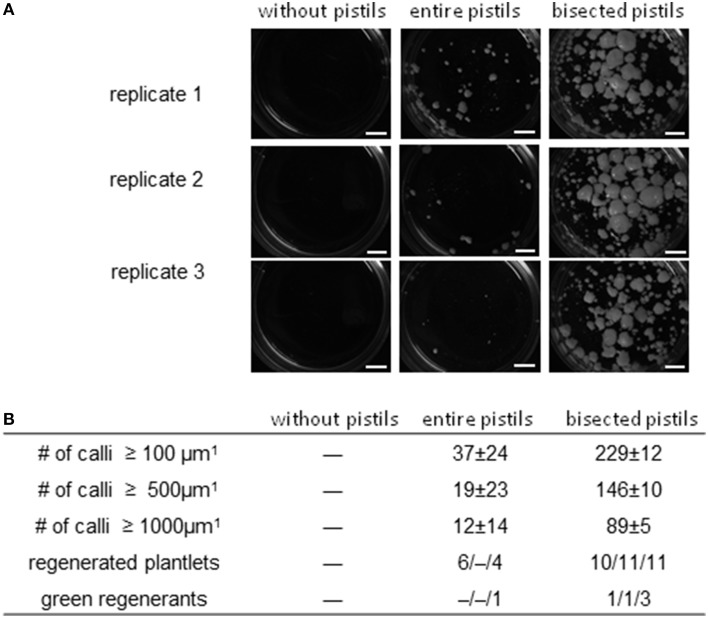
**The effect of entire and bisected pistils on embryogenic pollen development in the recalcitrant cv. “Golden Promise.”** Isolated microspores at an initial density of 5000 per mL medium were co-cultivated with either entire or bisected wheat pistils as compared to a negative control without nurse tissue. The microspores were kept in Millicell inserts to facilitate the observation of their response without being obscured by nurse tissue. The inserts were positioned in 3.5 cm Petri dishes containing a total volume of 2 mL medium, while the nurse tissue was kept in the medium portion outside the inserts. Three entire pistils or parts thereof were used per mL medium. **(A)** The formation of embryogenic calli after 4 weeks of pollen culture. Bar size = 2 mm. **(B)** Numbers of embryogenic calli obtained in three size classes (data shown in the form mean ± SD) and plantlet regeneration (data shown for total and green regenerants as single values from three different biological replicates).

Barley cv. “Golden Promise” is recalcitrant with respect to pollen embryogenesis (Coronado et al., 2005). When microspores isolated from cold-treated spikes were cultivated at a density of 5000 per mL without any nurse tissue, no callus formation ensued, but in the presence of uncut pistils, a small number of embryogenic calli of diameter ≥500 μm did form (Figure 4A). In all (across three biological replicates), 10 plantlets (0/4/6) were regenerated, of which only one (10%) was non-albino. When the nurse tissue consisted of longitudinally bisected pistils, the number of calli of diameter ≥100 μm formed was 229 ± 12 (six-fold increased), of which over half had a diameter of ≥500 μm. This treatment yielded a mean of 10–11 plantlets per replicate, of which 15.6% were green. The latter were successfully established in soil, constituting a five-fold improvement over the use of uncut pistils (Figure 4B).

### The identification of the molecular size range of the stimulatory compound(s) and its stability

**Figure 5:**
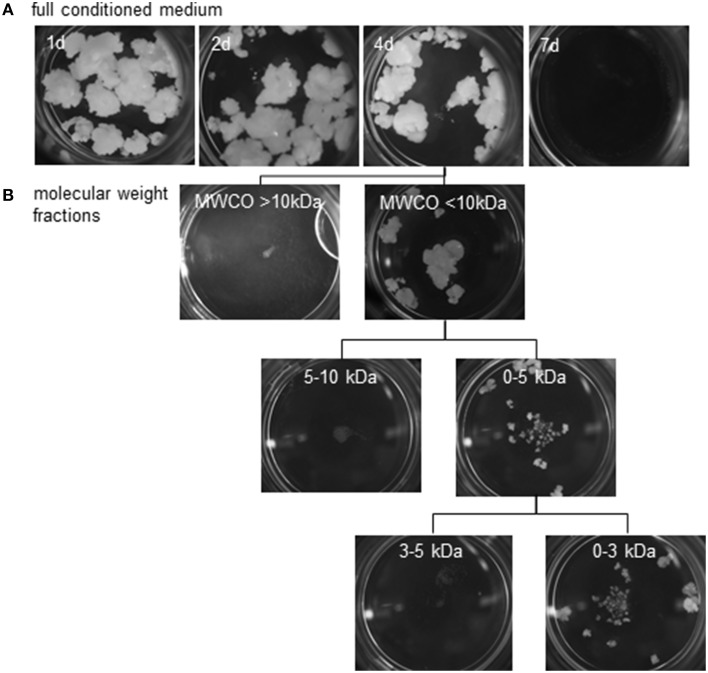
**Reduction of possible feeder compounds using size exclusion**. Embryogenic callus formation from populations of ca.150 immature pollen grains of cv. “Igri” supplemented at various intervals by **(A)** fresh pistil-preconditioned medium (intervals of medium exchange are given on the individual photographs recorded after 4 weeks of culture), **(B)** various size fractions of an extract of pistil-conditioned medium. A 4 day interval was sufficient to stimulate embryogenic development. The feeder effect is shown to be due to a compound(s) of size below the molecular weight cut-off of <3 kDa. MWCO: Molecular weight cut-off.

Pistil-preconditioned nutrient medium was only able to promote barley pollen embryogenesis when regularly refreshed. When an inductive medium was replaced only every 7 days, embryogenic development was not supported, whereas replacement every 4 days was effective (Figure 5A). As expected, however, embryogenic pollen development was much reduced as compared to continuous co-cultivation of bisected pistils. The composition of inductive medium was fractionated into two molecular size fractions, which were then added one by one to a non-inductive (low density, no nurse tissue) pollen culture. Stimulation of pollen embryogenesis was only induced by the presence of the smaller sized fraction which passed the molecular weight cut-off of ≤10 kDa. Further fractionation showed that the effect was due to a compound(s) of size below the molecular weight cut-off of ≤3 kDa (Figure 5B). The stimulatory effect of adding this size-fraction was lower than that directly provided by the cut pistils, conditioned medium or the ≤10 kDa fraction, which can be attributed to the expected loss of a substantial proportion of the effective signal molecules owing to the fractionation procedure.

## Discussion

The presence of immature pistils as a nurse material is known to stimulate *in vitro* embryogenic development of immature pollen in barley (Köhler and Wenzel, 1985; Li and Devaux, 2001; Lu et al., 2008), wheat (Puolimatka et al., 1996; Broughton, 2008), and other monocotyledonous (Szarka et al., 2001) and also dicotyledonous crops (Huang et al., 1990). Particularly in wheat, the co-cultivation of pistils has proved to be a critical component for doubled haploid production from isolated microspores (Hu and Kasha, 1997; Zheng et al., 2002).

The principle of pistil co-cultivation was subjected to a detailed investigation in the present study. A considerable challenge in doing so has been that not only dissected pistils, but also embryogenic barley pollen themselves have the capability of serving as highly effective nurse material. For instance, the co-cultivation of embryogenic barley pollen facilitated the establishment of embryogenesis and plant regeneration from isolated barley and wheat zygotes (Holm et al., 1994; Kumlehn et al., 1998) as well as follow-on investigations on the individual developmental fate of isolated zygotes and parthenogenetic egg cells of wheat (Kumlehn et al., 1999, 2001). In the present study, the stimulation induced by the nurse material was decoupled from that generated endogenously within the population of pollen grains by a careful manipulation of the culture density of the immature pollen grains. The micro-culture system developed for this purpose can be used to readily assess the benefit of different nurse tissues, extracts or specific compounds.

One of the drivers of the notion of developing such a micro-culture system was the suggestion that the stigma and style contain plentiful arabinogalactan proteins (Gane et al., 1995), a class of compounds which has been shown to enhance the processes of somatic embryogenesis in carrot (Van Engelen and de Vries, 1992; Van Hengel et al., 2001) and embryogenic pollen development in wheat (Letarte et al., 2006). However, the provision of stigma was less beneficial than that of bisected pistils or other parts thereof (Figure 2). As a nurse tissue, the chalazal part of the pistil has proved to be significantly superior to the basal, micropylar part of the pistil, implying that the relevant signaling molecules are more abundant in the former. The order of efficacy observed (bisected pistil < chalazal part of the pistil < ovary) suggests that the release of these signals is in some way dependent on if the pistil is cut, rather than on the particular tissue of pistils provided as a nurse tissue. The ineffectiveness of the ovules may reflect their inability to survive for an extended time under the given conditions *in vitro*. Although it was not possible to identify which specific part of the pistil was the source of the stimulatory signal (Figure 2B), embryogenic callus formation was considerably enhanced by cutting the pistils. The most likely explanation of this somewhat surprising finding is that damaging the pistil accelerated the secretion of the signaling molecules, although it is also conceivable that an upsurge in the production of signaling molecules forms part of the wounding response.

Although the supply of pistil-preconditioned medium was effective in stimulating pollen embryogenesis, the medium did need refreshing at least every 4 days, suggesting that the effective compound(s) was either rather labile or was consumed by the pollen. In contrast, according to Köhler and Wenzel (1985), the one-off provision of pistil-conditioned medium produces a detectable enhancement. Given however that in the latter study, the population density of the pollen was sufficient to self-induce embryogenesis, it is considered likely that even a comparatively short period of extra stimulation could have generated a detectable enhancement in embryogenic development. The stimulatory effect of the nurse pistils must have been exerted by a compound(s) below the molecular weight cut-off of 3 kDa, thereby excluding a number of potential candidates. These include both arabinogalactan proteins as well as chitinases that have been suggested to stimulate embryogenic development (De Jong et al., 1992; Letarte et al., 2006). What remain as potential viable candidates are various peptides such as the phytosulfokines or those identified in the nutrient medium of EPCs of rapeseed (Boutilier et al., 2005), phytohormones as suggested by Köhler and Wenzel (1985), polyamines, sugar derivatives and other metabolites. Identification of the signaling molecule(s) may help to define the identity of the relevant signaling pathway, but even if not, it could conveniently be used in pure form as an additive to promote whole plant regeneration from *in vitro* cultured immature pollen grains, and perhaps from other types of plant cell culture as well. Specifying the functionality of particular fractions or candidate molecules will be materially facilitated by the micro-culture system described in this communication.

A beneficial outcome of testing various pistil parts was that a practical level of embryogenic development and green plant regeneration was achievable from the pollen of the recalcitrant cv. “Golden Promise.” Extrapolating from an admittedly small-scale experiment suggests that ca. 15 non-albino regenerant plants could be derived from the pollen harvested from a single barley spike (ca. 10^5^ grains); such a level of yield would be well-acceptable in the context of doubled haploid based research and breeding. For comparison, after optimization of culture conditions for seven recalcitrant barley cultivars involving the co-cultivation of (uncut) wheat pistils, Li and Devaux (2001) obtained about 15 regenerant plants per spike on average, out of which ca. 16% were green, which is well-comparable with the result of the present study in cv. “Golden Promise” when uncut pistils were used as nurse tissue. In addition, these results are on a par with what was previouly achieved in cv. “Golden Promise” under the same inductive conditions (4 weeks cold treatment of spikes) as in the present study along with standard culture density and co-culture of uncut wheat pistils (Coronado et al., 2005). However, the efficiency of total and green plant regeneration achieved here using bisected pistils was not only superior to the directly compared use of uncut pistils, but also exceeded the result obtained by Li and Devaux (2001) also with regards to the best performing one amongst the recalcitrant cultivars tested in their study.

The occurrence of albino plantlets is a particular problem in EPCs of grass species and is thought to be associated with the impact of stress required to induce pollen embryogenesis (Ankele et al., 2005). Owing to the lack of chlorophyll, such individuals are not capable of being photosynthetically active and thus cannot be established in soil. Whereas, the efficiency of embryogenic development and total plant regeneration was improved by the principle of bisecting the pistils used for co-cultivation, the ratio of green plant formation was at best slightly enhanced as compared to the use of uncut pistils.

## Summary and perspective

Signal molecules secreted into the nutrient medium either by the cultivated pollen themselves or by co-cultivated barley or wheat pistils are indispensible for the development of EPCs of barley, which holds undoubtedly also true for any other plant species. In the absence of heterologous nurse tissue, there is no embryogenic development at a culture density of ≤1600 immature barley pollen grains per mL medium. While the cellular origin of the effective compound(s) secreted from co-cultivated wheat pistils is not specific to any tested pistil part, the increased recovery of calli during treatment with bisected pistils indicates improved release of those molecules from the explants and may provide a useful tool for plant breeding and research using haploid technology in barley, as was exemplified in the present study using the cv. “Golden Promise,” and probably in other plant species as well. In addition, effective nurse systems will be particularly useful in EPCs subjected to genetic manipulations such as induced mutagenesis and transformation, since the cultured pollen grains often show a high mortality owing to the impact of the applied chemical, radiation, particle bombardment or agro-inoculation. As a consequence, only a subpopulation of at best intermediate culture density is left behind. In such situations, a nurse system may compensate for the lost self-feeding effect which is only ensured at higher culture density.

This study unambiguously narrowed down the size of candidate compounds to a molecular weight cut-off of 3 kDa, and we demonstrate that the effectiveness of the secreted molecules was only transitory. This information is valuable for future studies aiming to identify particular molecules that support embryogenic development in plant cell cultures. In the same context, the micro-culture system developed in this study for low-density cultures holds great promise for the assessment of stimulatory effects of other nurse tissues, extracts, knowledge-based candidate compounds or those contained in large molecule libraries. Moreover, the highly effective nurse system established may greatly facilitate the culture, observation and manipulation of particularly sensitive cells and tissues, protoplasts as well as single cells.

## Methods

### Donor plants, stress treatment, and the isolation of immature pollen

Grains of the winter barley cultivar “Igri” and the spring barley cultivar “Golden Promise” were germinated under a 16 h photoperiod and a day/night temperature of 14/12°C. Two week-old seedlings of cv. “Igri” were then vernalized for 8 weeks by exposure to a 9 h photoperiod at 2°C; finally, the seedlings of both cultivars were further grown under 16 h photoperiod and a day/night temperature of 18/14°C. Immature pistils were harvested from the wheat cultivar “Bob White” at a stage when the pollen was bi-cellular. Immature barley spikes were detached when the majority of pollen was right before entering pollen mitosis I, held at 4°C for about 4 weeks, then chopped into 2–3 cm long segments prior to homogenization in 20 mL cold 0.4 M mannitol for 20 s. The homogenate was filtered through 100 μm nylon mesh and the particulate matter re-blended in 10 mL 0.4 M mannitol and re-filtered. The combined filtrate was centrifuged (100 × g, 4°C, 10 min), the supernatant was discarded and the pellet re-suspended in 5 mL 0.55 M maltose, which was then over-layered with 1.5 mL 0.4 M mannitol. After a second centrifugation, the interphase, in which most of the immature pollen grains had been concentrated, was withdrawn and suspended in 20 mL 0.4 M mannitol. The density of pollen grains present was estimated using a haemocytometer and the suspension centrifuged once more as above. The supernatant was discarded and the pellet re-suspended in sufficient KBP medium (Kumlehn et al., 2006) to obtain a final density of approximately 10,000 pollen grains per mL, if not specified otherwise. The exact density was estimated from a direct count of the number of pollen grains present in five separate 1 μL aliquots prior to the establishment of cultures described below.

### *In vitro* pollen culture

A 12 mm Millicell Culture Plate Insert (Merck Millipore Darmstadt, Germany) with a hydrophilic, permeable and transparent PTFE membrane (0.4 μM pore size) in place of a solid bottom plate was positioned inside a 3.5 cm Petri dish containing 2 mL KBP medium, of which 200 μL was pipetted into the insert. Using this culture system, test cells can be grown separated from nurse tissue which itself is co-cultivated in the same medium but outside the insert, while signal molecules are allowed to freely move through the membrane between both partial volumes of the culture medium.

Adequate aliquots of the immature pollen suspension of cv. “Igri” were added to the inserts to establish culture densities of 75–6400 immature pollen grains per mL with respect to the total volume of medium in the Petri dish. The nurse material was added to the Petri dish outside the Millicell insert, the dishes were sealed with Parafilm and then held at 24°C in the dark.

EPCs of cv. “Igri” to be used as nurse tissue were initially prepared at a culture density of 100,000 microspores per 3.5 cm Petri dish containing 1 mL KBP medium. After 1–2 weeks of preculture, the medium was replaced by 1.8 mL fresh medium and a Millicell insert containing another 200 μL medium along with the test pollen was positioned into the dish. The resultant density of the EPC used as nurse tissue was 50,000 per mL with respect to the total volume of medium in the dish.

Immature pollen grains extracted from cv. “Golden Promise” were cultured at a density of 5000 per mL KBP medium. This was achieved by culturing 10,000 isolated microspores per Millicell insert, while the total volume of medium per 3.5 cm Petri dish was 2 mL (as detailed above). After 4 weeks of culture on a shaker at 65 rpm, any calli which had developed were placed on K4NB medium (Kumlehn et al., 2006) in the dark at 26°C for 1 week, then brought into the light (16 h per day). After a further 3 weeks, the differentiated plantlets were counted and individually transferred to 6-cm pots containing soil substrate. These pots were placed in a tray covered by a transparent hood to maintain saturated humidity environment for about 2 weeks. Further growth conditions were as described above for the donor plants.

For the statistical analysis of embryogenic pollen development, a Student-Newman-Keuls test was performed across six biological replicates per treatment using SigmaStat 3.0 software (Systat Software GmbH, Germany).

### Use of conditioned medium and the identification of maximum molecular weight of the stimulating molecule(s)

KBP medium (Kumlehn et al., 2006) conditioned by cultivation of 10 bisected wheat pistils per mL for 7 days was used as a supplement to support embryogenic pollen development without direct co-cultivation of nurse tissue. In EPCs without nurse tissue, conditioned media were refreshed at intervals of 1–7 days over a period of 28 days. In addition, media pre-conditioned by pistil cultivation were subjected to molecular size fractionation, which was effected using polyethersulfon Vivaspin 6 size exclusion concentrators with molecular weight cut-off levels of 10, 5, and 3 kDa (Sartorius AG, Germany). The retentate was solubilized in fresh KPB medium. The filtrate and solubilized retentate fractions were filter-sterilized through a 0.2 μm filter before being tested for their effect on embryogenic pollen development in cv. “Igri” using a culture density of 75 per mL.

## Author contributions

JK, HM, and RL designed the research, RL performed the experiments, JK, RL, HM, and SF analyzed the data, RL and JK wrote and SF and HM critically edited the manuscript.

### Conflict of interest statement

The authors declare that the research was conducted in the absence of any commercial or financial relationships that could be construed as a potential conflict of interest.

## Supplementary material

Figure S1 **The effect of using whole or partial wheat pistils as a nurse material on embryogenic pollen development in cv. “Igri” after 7, 14, and 24 days of culture**. Representative examples of cultures involving as nurse material. P, pistil; St, stigma; Ovr, ovary; mOvr, mycropylar ovary half; cOvr, chalazal ovary half; cP, cross-bisected pistil; lP, longitudinally bisected pistil; lP-Ovu, longitudinally bisected pistil without ovule; Ovu, ovule; lOvu, longitudinally bisected ovule; EPC, embryogenic pollen culture of cv. “Igri” precultured for 1–2 weeks and used as nurse tissue at a density of 50,000 pollen grains per mL as compared with noNT: no nurse tissue as negative control. Bar size for seven and 14 days old cultures: 100 μm; bar size for 24 days old cultures: 2 mm.

Table S1 **The effect of using whole or partial wheat pistils as a nurse tissue on embryogenic pollen development in cv. “Igri” at low culture density (75 isolated microspores per mL) as assessed after 4 weeks of culture**. Embryogenic calli of diameter **(A)** ≥100 μm, **(B)** ≥500 μm, and **(C)** ≥1 mm. noNT, no nurse tissue; P, pistil; St, stigma; Ovr, ovary; mOvr, mycropylar ovary half; cOvr, chalazal ovary half; cP, cross-bisected pistil; lP, longitudinally bisected pistil; lP^−Ovu^, longitudinally bisected pistil without ovule; Ovu, ovule; lOvu, longitudinally bisected ovule; EPC, embryogenic pollen culture of cv. “Igri” precultured for 1–2 weeks and used as nurse tissue at a density of 50,000 pollen grains per mL.

Table S2 **The effect on embryogenic pollen development in cv. “Igri” of varying the number of co-cultivated, longitudinally bisected pistils (lP, numbers used per mL medium are given)**. An embryogenic pollen culture (EPC) of cv. “Igri” was used as a positive control at a population density of 50,000 pollen grains per mL. The numbers of embryogenic calli of diameter **(A)** ≥100 μm, **(B)** ≥500 μm, **(C)** ≥1 mm were assessed following 4 weeks of culture. noNT: no nurse tissue.
