# Supplementary material for: The low molecular weight fraction of compounds released from immature wheat pistils supports barley pollen embryogenesis
Source: Front Plant Sci. 2015 Jul 7;6:498. doi: 10.3389/fpls.2015.00498 (PMC4493395; doi:10.3389/fpls.2015.00498)
Supplement: Figure S1 — The effect of using whole or partial wheat pistils as a nurse material on embryogenic pollen development in cv. “Igri” after 7, 14, and 24 days of culture. Representative examples of cultures involving as nurse material. P, pistil; St, stigma; Ovr, ovary; mOvr, mycropylar ovary half; cOvr, chalazal ovary half; cP, cross-bisected pistil; lP, longitudinally bisected pistil; lP-Ovu, longitudinally bisected pistil without ovule; Ovu, ovule; lOvu, longitudinally bisected ovule; EPC, embryogenic pollen culture of cv. “Igri” precultured for 1–2 weeks and used as nurse tissue at a density of 50,000 pollen grains per mL as compared with noNT: no nurse tissue as negative control. Bar size for seven and 14 days old cultures: 100 μm; bar size for 24 days old cultures: 2 mm. [file Presentation1.PPTX]

## Slide 1
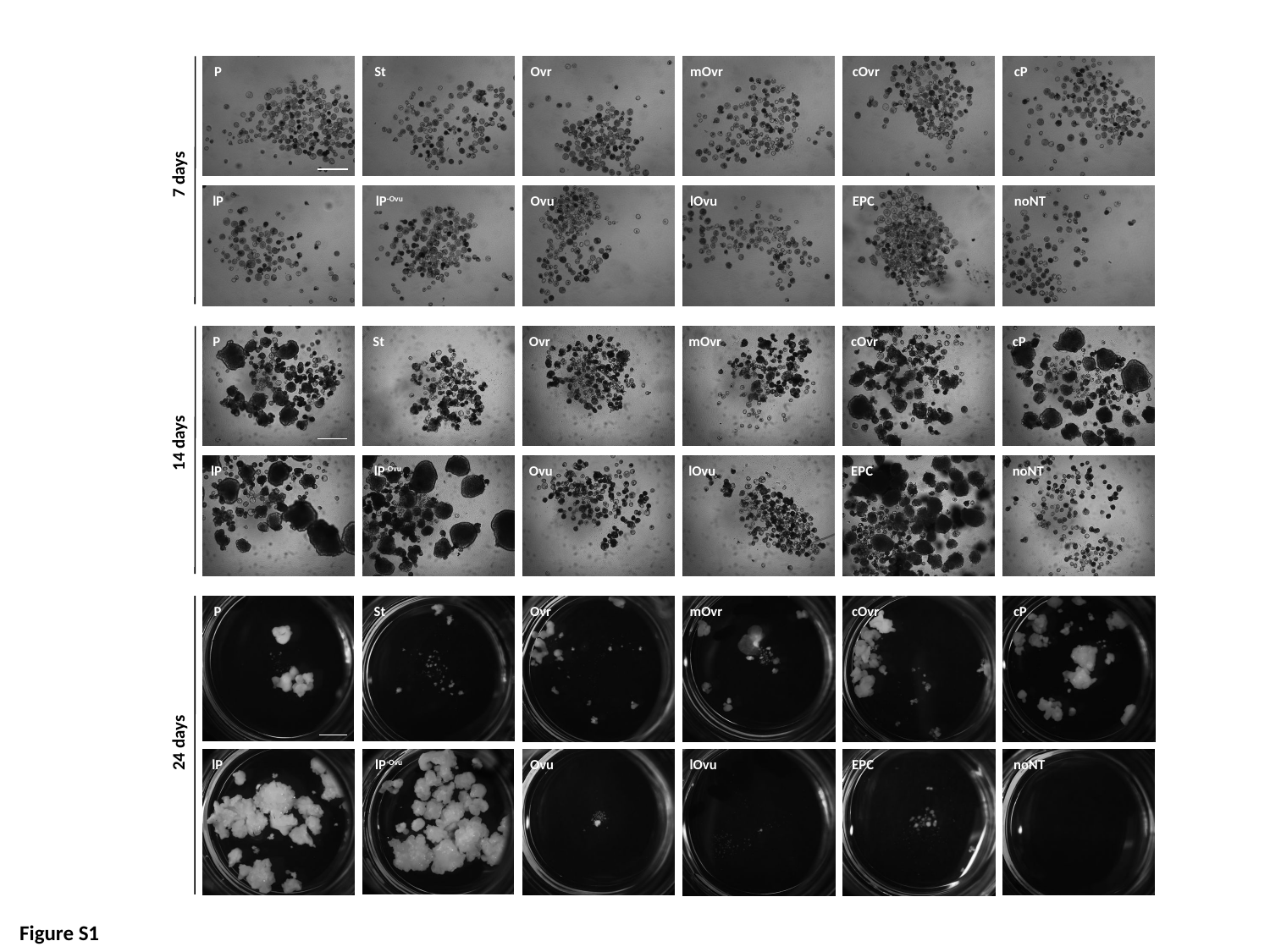

P
St
Ovr
mOvr
cOvr
cP
lP
lP-Ovu
Ovu
lOvu
EPC
noNT
7 days
P
St
Ovr
mOvr
cOvr
cP
lP
lP-Ovu
Ovu
lOvu
EPC
noNT
14 days
P
St
Ovr
mOvr
cOvr
cP
lP
lP-Ovu
Ovu
lOvu
EPC
noNT
24 days
Figure S1
